# Anti-inflammatory effects of *Radix Gentianae Macrophyllae *(*Qinjiao*), *Rhizoma Coptidis *(*Huanglian*) and *Citri Unshiu Pericarpium *(*Wenzhou migan*) in animal models

**DOI:** 10.1186/1749-8546-3-10

**Published:** 2008-09-02

**Authors:** Kyoung Soo Kim, Hae In Rhee, Eun Kyung Park, Kiwon Jung, Hyo Jin Jeon, Ji-Hong Kim, Hunseung Yoo, Chang-Kyun Han, Yong-Baik Cho, Chun Jeih Ryu, Hyung In Yang, Myung Chul Yoo

**Affiliations:** 1 East-West Bone and Joint Research Center, Kyung Hee University Neo Medical Hospital, 149 Sangil-dong, Gangdong-gu, Seoul 134-090, South Korea; 2 Life Science Research Center, SK Chemicals, 600 Jungja-1-dong, Changan-Ku, Suwon-Si, Kyoungki-Do 440-745, South Korea; 3 Central R&D, Ahngook Pharm., Iui-Dong Yeongtong-gu, Suwon-Si, Kyoungki-Do, South Korea; 4 Central Research Institute, Whan In Pharm., Co., Ltd. Iui-Dong, Yeongtong-gu, Suwon-Si, Kyoungki-Do, South Korea; 5 Department of Bioscience and Biotechnology, Sejong University, Seoul 143-747, South Korea; 6 Department of Internal Medicine, Kyung Hee University Neo Medical Hospital, 149 Sangil-dong, Gangdong-gu, Seoul 134-090, South Korea; 7 Department of Orthopedic Surgery, Kyung Hee University Neo Medical Hospital, 149 Sangil-dong, Gangdong-gu, Seoul 134-090, South Korea

## Abstract

**Background:**

KHU14, an ethanolic extract of *Radix Gentianae Macrophyllae *(*Qinjiao*), *Rhizoma Coptidis *(*Huanglian*) and *Citri Unshiu Pericarpium *(*Wenzhou migan*) was tested for its anti-inflammatory effects.

**Methods:**

Three out of 20 herbs were found to have anti-inflammatory effects. The formulation of these herbs, i.e. KHU14 was tested for croton oil-induced ear edema, carrageenan-induced paw edema, acetic acid-induced capillary permeability, cotton pellet and delayed type hypersensitivity.

**Results:**

KHU14 exhibited anti-inflammatory effects in animal models of acute and chronic inflammation. The anti-inflammatory activity of KHU14 observed was comparable to that of celecoxib. KHU14 inhibited the production of NO and PGE_2 _in LPS/IFN-gamma-stimulated peritoneal macrophages, and reduced edema and the amount of infiltrated cells in animal models.

**Conclusion:**

KHU14 exhibited anti-inflammatory effects as demonstrated in typical immunological tests for anti-inflammation *in vitro *and *in vivo*.

## Background

Herbal extracts from traditional Chinese medicine can be formulated to develop novel herbal medicines as potent as synthetic medicines [1-3]. We investigated *in vitro *anti-inflammatory properties of 20 medicinal herbs used in Chinese medicine in order to develop a new herbal formulation to treat inflammation. Three herbs, namely *Radix Gentianae Macrophyllae *(*Qinjiao*) [4,5], *Rhizoma Coptidis *(*Huanglian*) [6] and *Citri Unshiu Pericarpium *(*Wenzhou migan*) [7,8], demonstrated anti-inflammatory effects in various experimental models. The primary ingredient in *Radix Gentianae Macrophyllae *is gentiopicroside which was shown to have anti-inflammatory effects in a murine model of hepatic injury [9]. Berberine, which has strong anti-inflammatory effects [10-12], is a major active constituent of *Rhizoma Coptidis*. Hesperidin [13] and nobiletin [14], both of which exhibit anti-inflammatory effects, are the active ingredients in *Citri Unshiu Pericarpium *[15,16]. Our *in vitro *screening and other available information suggests that these three herbs have potential anti-inflammatory effects. Therefore, these three herbs were selected for a formulation, i.e. KHU14. The present study tests the anti-inflammatory actions of KHU14 in several animal models of inflammation.

## Methods

### Materials

Carboxymethyl cellulose (CMC), dexamethasone, olive oil, 4-ethoxymethylene-2-phenyloxazolone, acetone, carrageenan, croton oil, Evans blue, and Griess regent (1% sulfanilamide and 0.1% N- [napthyl] ethylenediamine dihydrochloride in 2.5% H_3_PO_4_) were purchased from Sigma (USA). Celecoxib (capsules) was purchased from Pfizer Pharmaceuticals (Korea). ELISA kits for interleukin-2 and interferon-γ and the immunoassay kit for PGE_2 _were purchased from R&D Systems (USA). RPMI 1640 (Gibco, UK) and DMEM (Invitrogen, UK), antibiotic-antimycotic solution (Gibco, UK) and fetal bovine serum (FBS, CAMBREX, USA) were used as media for cell culture. The 20 herbs used in the present study were purchased from Kyung Hee Oriental Medical Hospital.

### Animals

Female *BALB/c *mice (5–6 weeks old, 16–18 g) and male ICR mice (5–6 weeks old, 16–18 g) were obtained from Orient Co Ltd (Korea). Male Wistar rats (5–6 weeks old, 200–300 g) were obtained from SLC Co Ltd (Japan). All animals were kept in plastic cages at 21–24°C under a 12 hour light/dark cycle and were given free access to pellet food and water. The mice were fed with 200 μl of the extract solution and the rats were fed with 2 ml of the same. This study complied with the internationally accredited guidelines and ethical regulations on animal research.

### Preparation of plant extracts

Powdered *Radix Gentianae Macrophyllae*, *Rhizoma Coptidis *and *Citri Unshiu Pericarpium *were obtained from Kyung Hee Oriental Medical Hospital (South Korea). The powders of these herbs (200 g each) were mixed by blending and then extracted twice with 50% ethanol (1800 ml) at 80°C for 4 hours. The combined ethanolic extracts were filtered and concentrated in a rotary evaporator at 40°C. The yield (59.5 g), code named KHU14 (KHU referring to Kyung Hee University), was then dissolved in 0.5% carboxylmethyl cellulose (CMC) solution (0.5 g CMC in 100 ml of distilled water) for the subsequent *in vivo *experiments. The voucher specimens of the plants used in this study were stored in the department herbarium for future reference.

### Measurement of cell viability

Cell viability was assessed by the 3'-(4,5-dimethylthiazole-2yl)-2,5-diphenyltetrazolium bromide (MTT) assay. RAW264.7 cells (1 × 10^4 ^cells/well) were seeded in triplicates of 24-well plates and cultured in 1 ml of Dulbecco's Modified Essential Medium (DMEM) containing 10% fetal bovine serum (FBS) overnight. After treated with KHU14 for one hour, cells were stimulated with 1 μg/ml of LPS for 72 hours and MTT (0.5 mg/ml) was added in the third hour. After the removal of the medium and the addition of 500 μl of DMSO to the well, the optical density (OD) absorbance was measured at 570 nm.

### Western blot anlysis

RAW264.7 cells cultured (1 × 10^6 ^cells) in 60 mm dishes were serum-starved overnight. After the cells were treated with KHU14 for 1 hour, the cells were stimulated by LPS (1 μg/ml) for 24 hours. The cells were subsequently washed twice in PBS and treated with 50 μl of lysis buffer (20 mM Tris-Cl [pH 8.0], 150 mM NaCl, 1 mM EDTA, 1% Triton X-100, 20 μg/ml chymostatin, 2 mM PMSF, 10 μM leupeptin, and 1 mM 4-(2-aminoethyl) benzenesulfonyl fluoride [AEBSF]). The samples were separated with 12% SDS-PAGE and were then transferred to Hybond-ECL membranes (Amersham, USA). The membranes were first blocked with 6% nonfat milk dissolved in TBST buffer (10 mM Tris-Cl [pH 8.0], 150 mM NaCl, 0.05% Tween 20). The blots were then probed with various rabbit polyclonal antibodies for iNOS, COX-2 and β-actin (Cell Signaling Technology, USA) diluted 1:1000 in TBS for 2 hours and incubated with 1:1000 dilutions of goat anti-rabbit IgG secondary antibody coupled with peroxidase. The blots were developed with the ECL method (Amersham, USA). For re-probing, the blots were incubated in the stripping buffer (100 mM 2-mercaptoethanol, 2% SDS, 62.5 mM Tris-HCl [pH 6.7]) at 50°C for 30 minutes with occasional agitation.

### Preparation of activated peritoneal macrophages from mice

Resident macrophages were obtained by peritoneal lavage according to a previously published method [17]. Briefly, the mice were injected intraperitoneally with 1 ml of Brewer thioglycollate medium (3%); and peritoneal fluids were harvested after three days. The peritoneal exudates were centrifuged at 2000 rpm (931 × *g*, Allegra™ X-12R Centrifuge, Beckman Coulter, USA) for 5 minutes at 4°C. The cell pellets were washed twice with DMEM containing 10% FBS, 100 U/ml penicillin, 100 mg/ml streptomycin. The washed cells were stimulated with lipopolysaccharide (LPS, 1 μg/ml) and IFN-γ (1 ng/ml) for 96 hours on 96-well plates (2 × 10^5 ^cells in 200 μl of medium per well) for the nitric oxide (NO) assay, and 24 hours on 24-well plates (each well contains 1 × 10^6 ^cells in 1 ml of medium) for the prostaglandine E_2 _(PGE_2_) assay.

### NO and PGE_2 _assays

Total NO production may be measured by nitrite assay as NO is rapidly converted to nitrite and nitrate water. Briefly, 100 μl of the culture supernatant was incubated at room temperature for 10 minutes with 100 μl of Griess reagent (1% sulfanilamide, 0.2% N-(1-naphthyl) ethylenediamine dihydrochloride in 2.5% H_3_PO_4_). The OD was measured at 570 nm and nitrite concentration was determined with a standard curve. We used an enzyme immunoassay kit to measure the PGE_2 _production in the culture supernatant following the manufacturer's instructions (R&D Systems, USA).

### Ear edema induced by croton oil

The inner surface of the right ear of the male ICR mice was treated with 20 μl of freshly prepared croton oil (2.5% in acetone). The left ear was treated with 20 μl of acetone as control [18]. The thickness of the ear edema was measured with an engineering gauge (Model H, Peacock, Japan) 4 hours after the application of the irritant. Sixty minutes prior to the induction of edema, KHU14 (400 mg/kg of body weight), celecoxib (100 mg/kg of body weight) and vehicle (0.5% CMC) were orally administered to three groups of animals which had fasted for four hours. Edema was measured as the difference between the thickness of the control ear and that of the ear treated with croton oil.

### Paw edema induced by carrageenan

Each of the male Wistar rats was injected with 0.1 ml of a freshly prepared suspension of carrageenan in saline (2.0 mg/ml) in the subplanta tissue of the right hind paw. An equal volume of saline was injected into the left hind paw as control. We made some modifications to the previously described murine paw edema model [19]. The volume of the paw up to the tibiotarsal joint was measured with a plethysmometer (Model 7140, Ugo Basile, Italy) one, two and four hour(s) respectively after the induction of inflammation. Edema was measured as the difference between the volume of the paw of the control and that of the paw injected with carrageenan. Sixty minutes prior to the induction of edema, KHU14 (400 mg/kg of body weight), celecoxib (100 mg/kg of body weight) and vehicle (0.5% CMC) were orally administered to three groups of animals which had fasted for 15 hours.

### Capillary permeability increase induced by acetic acid

Vascular permeability increase induced by acetic acid in the male ICR mice was determined following a modified Whittle method [20]. KHU14 (400 mg/kg of body weight), celecoxib (100 mg/kg of body weight) and vehicle (0.5% CMC) were orally administered to three groups of mice respectively. Thirty minutes after the administration, each mouse was intravenously injected (at the tail) with 0.1 ml of 4% Evans blue (10 μl/g of body weight, Sigma, USA) in saline. Fifteen minutes after the intravenous injection, each mouse was intraperitoneally injected with 0.1 M acetic acid (10 μl/g body weight). The mice were sacrificed by ether 20 minutes after the acetic acid injection. The viscera were exposed for one minute to drain the blood and were washed with saline over a Petri dish. The wash was poured into 10 ml volumetric flasks through glass wool. Each flask contained 10 ml of distilled water and 0.1 ml of a sodium hydroxide solution (0.1 M) was added to the flask to clear any turbidity caused by proteins. The OD was measured at 590 nm.

### Cotton pellet test

Two sterile cotton pellets (10 mg) were subcutaneously delivered into the dorsum of the male ICR mice anesthetized with ether. KHU14 (400 mg/kg of body weight), celecoxib (100 mg/kg of body weight) and vehicle (0.5% CMC) were orally administered to three groups of mice respectively once daily for seven days. The mice were sacrificed on the 7^th ^day by ether. The cotton pellets were removed, dried at 37°C for 24 hours and weighed. The results were expressed as the difference between the initial weight (10 mg) and the final dry weight of the cotton pellets.

### Delayed type hypersensitivity

Female *BALB/c *mice were sensitized by epicutaneously applying 25 μl of a mixture of acetone and olive oil (4:1) containing 2% 4-ethoxymethylene-2-phenyloxazolone on the shaved abdomen and thorax skin as described by Blaylock *et al*. [21]. KHU14 (400 mg/kg of body weight), dexamethasone (1 mg/kg of body weight) and vehicle (0.5% CMC) were orally administered to three groups of mice at one, three, and five days respectively after sensitization. One day after the last feed of test sample, all mice were challenged by applying 10 μl of 0.5% oxazolone in a mixture of acetone and olive oil (4:1) to the inner and outer surfaces of the right ear. The inhibitory effect of the test sample on the delayed type hypersensitivity (DTH) reaction was determined in comparison to the DTH reaction in 0.5% CMC-fed mice. The intensities of the DTH reaction were measured as the difference between the right ear thickness and the left ear thickness 24 hours after the 0.5% oxazolone challenge.

### Statistical analysis

The results are expressed as mean and standard deviation (SD). The statistical significance between groups was determined by ANOVA and by non-parametric Kruskal-Wallis test with the GraphPad Prism 4 software (GraphPad, USA). P values less than 0.05 were considered to be statistically significant.

## Results and discussion

### Selection of three herbal extracts from 20 herbs

To develop potent multiple herbal extracts with anti-inflammatory effects, we evaluated the *in vitro *anti-inflammatory effects of each herbal extract in terms of the production of NO in LPS and IFN-γ-stimulated peritoneal macrophages (Table 1). Out of the 20 herbs, three were selected and combined to form KHU14 according to their anti-inflammatory effects and traditional usage in Chinese medicine and their major active ingredients. KHU14 was evaluated for its anti-inflammatory effects against those of a single herb (Table 2).

**Table 1:** Effects of 20 single herbal extracts (100 μg/ml) on nitrite in mouse peritoneal macrophages stimulated by LPS and IFN-γ

	1	2	3	4	5	6*	7	8*	9	10	11	12	13	14	15*	16	17	18	19	20
mean (μM)	61.3	55.4	52.2	48.8	50.2	45.3	41.8	47.9	50	44.9	50.1	65.8	40.6	39.1	38.2	53.1	45.3	39.1	38.4	40.1
SD	1.7	2.7	1.5	2.2	2.9	2.8	3.5	2.1	3.6	2.8	2.1	4.3	2.7	3.5	2.3	1.8	2.1	3.6	2.7	3.8

1 *Sophora subprostrata*; 2 *Siegesbeckia pubescens*; 3 *Angelica acutiloba*; 4 *Vitex rotundifolia*; 5 Anemarrhena asphodelodies; *6 *Gentiana macrophylla; *7 *Poria cocos*; *8 *Citrus unshiu*; 9 *Eucommia ulmodies*; 10 *Polygonatum sibiricum*; 11 *Cinnamomum cassia*; 12 *Chaenomels sinensis*; 13 *Atractylodes lancea*; 14 *Aralia cordata*; *15 *Coptis chinensis*; 16 *Clematis mandshurica*; 17 *Phellodendron amurensis*; 18 *Scutellaria baicalensis*; 19 *Cimicifuga heracalensis*; 20 *Pueraria thunbergiana*

*: Selected herbs for the KUH14 formulation

**Table 2:** Effects of KHU14 and its herbal components (100 g/ml) on nitrite in mouse peritoneal macrophages stimulated by LPS and IFN-γ

	KHU14	*Radix Gentianae Macrophyllae*	*Rhizoma Coptidis*	*Citri Unshiu Pericarpium*
Mean (μM)	40.2	49.2	41.9	50.7
SD	2.1	3.8	3.1	3.5

### Effects of KHU14 on cell viability and iNOS and COX-2 expression in RAW264.7 cells

**Figure 1:**
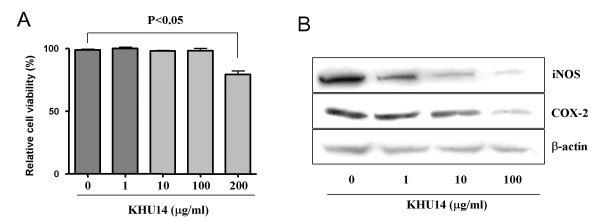
**Effects of KHU14 on cell viability and iNOS and COX-2 expression in RAW264.7 cells**. (A) Cell viability. (B) Western blot. RAW264.7 cells were treated with 0, 1, 10, 100, 200 μg/ml of KHU14 dissolved in DMSO one hour before stimulated with LPS (1 μg/ml) for 72 hours for measurement of cell viability. For western blot analysis, cells were stimulated with LPS for 24 hours in the presence of KHU14. The results are expressed as mean and SD.

To identify the toxic effect of KHU14, we tested its effects on the viability of a murine macrophage cell line (RAW264.7) with 0.025% trypan blue dye exclusion method. The exposure of the cells to KHU14 (1–100 μg/ml) for 72 hours showed no significant adverse effect on the cell viability, while after exposure to KHU14 (200 μg/ml) for 72 hours, the cell viability was reduced to 80% of that of the control (Figure 1A). In addition, KHU14 (100 μg/ml) inhibited the expression of iNOS and COX-2 in an LPS-activated RAW264.7 cell line without causing cytotoxicity (Figure 1B).

### In vitro effects of KHU14 on NO and PGE_2 _in peritoneal macrophages

**Figure 2:**
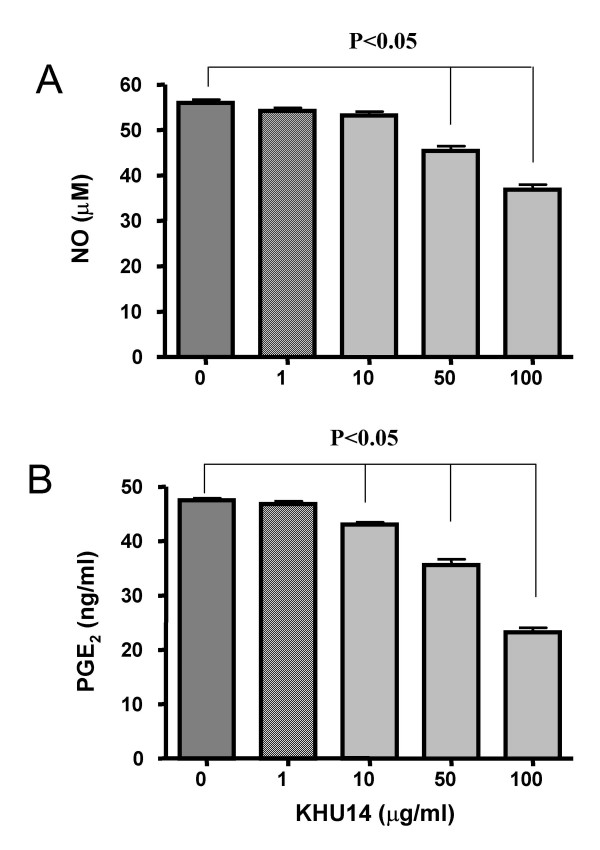
**Effects of KHU14 on the NO and PGE_2 _production in mouse peritoneal macrophages**. The cells were treated with 0, 1, 10, 50 and 100 μg/ml of KHU14 dissolved in DMSO respectively 30 minutes before stimulated with LPS (1 μg/ml) + IFN-γ (1 ng/ml) for 24 hours. The supernatants were collected and used for the measurement of the amount of (A) NO and (B) PGE_2_. The results are expressed as mean and SD.

Macrophages play a central role in the overproduction of pro-inflammatory cytokines and inflammatory mediators such as NO (Figure 2A) and PGE_2 _(Figure 2B). KHU14 (1–100 μg/ml) significantly inhibited LPS/IFN-γ-induced NO and PGE_2 _production in peritoneal macrophages in a dose-dependent manner. KHU14 (100 μg/ml) inhibited NO and PGE_2 _production by 27% and 49% respectively.

### In vivo effects of KHU14 on acute inflammation

We employed three animal models of acute inflammation to evaluate the anti-inflammatory effects of KHU14: (1) inhibition of croton oil-induced ear edema in mice; (2) carrageenan-induced paw edema in rats; and (3) acetic acid-induced permeability test in mice.

**Figure 3:**
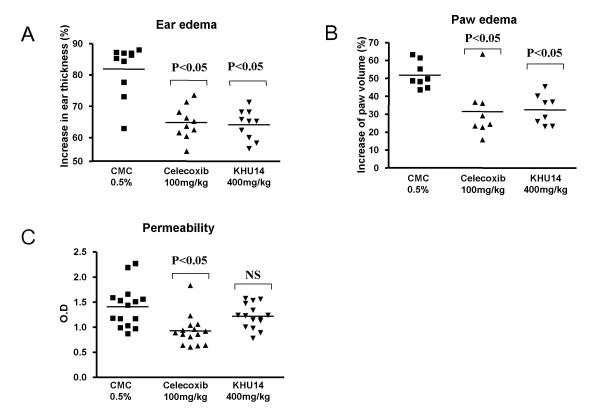
**Anti-inflammatory effects of KHU14 on animal models of acute inflammation**. (A) Ear edema in mice (n = 10). (B) Paw edema in rats (n = 8). (C) Capillary permeability test (n = 15). The mice and rats were orally fed with KHU14 (400 mg/kg of body weight) and celecoxib (100 mg/kg of body weight). The control group received 0.5% CMC orally. NS: not significant.

In the croton oil-induced ear edema model for testing the topical anti-inflammatory effects of KHU14, the control group which received 0.5% CMC increased 81.8% in ear thickness. The KHU14 and celecoxib groups increased 64.8% and 64.1% in ear thickness respectively. The results indicate that KHU14 and celecoxib reduced ear thickness by 20% (Figure 3A). In the carrageenan induced paw edema test, the paw volume increased 51.8% in the control group which received CMC, while it increased 31.4% and 32.3% in the celecoxib and KHU14 groups respectively. The results indicate that celecoxib and KHU14 reduced paw edema by 40% and 38% respectively (Figure 3B). In the acetic acid-induced permeability test, celecoxib group reduced permeability by 35%; KHU14, however, did not significantly reduce the permeability of Evan blue (Figure 3C).

### In vivo effects of KHU14 on chronic inflammation

**Figure 4:**
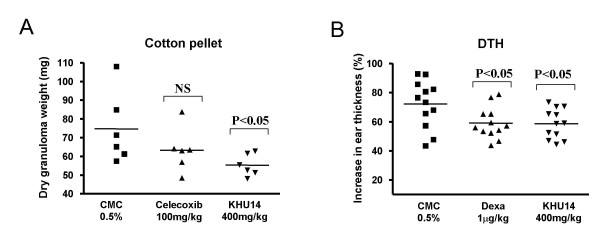
**Anti-inflammatory effects of KHU14 on a mouse model of chronic inflammation**. (A) Cotton pellet test (n = 6). (B) DTH (n = 12). The mice were orally fed with KHU14 (400 mg/kg of body weight) and celecoxib (100 mg/kg of body weight) for cotton pellet test and dexamethasone (1 mg/kg of body weight) for DTH. The control group received 0.5% CMC orally. NS: not significant.

In the cotton pellet test, the dry cotton in CMC, celecoxib, and KHU14 groups weighted 74.7 mg, 63.3 mg, and 55.3 mg respectively (Figure 4A). KHU14 inhibited the infiltration of immune cells by 26% with statistical significance. In the oxazolone-induced DTH model, the right ear thickness of the control group increased 72.1%, while it increased 59.0% and 58.6% in the dexamethasone group and KHU14 group respectively. The results indicate that dexamethsone and KHU14 reduced ear swelling by 18% and 19% respectively (Figure 4B).

The results of the present study suggest that KHU14 has considerable potency in anti-inflammatory action and that it can be used as an anti-inflammatory agent to treat certain inflammatory diseases. To further determine its therapeutic effects, we will evaluate the toxicity of KHU14 *in vivo *and in animal models of diseases such as collagen-induced arthritis [22] and psoriasis-like skin diseases [23]. Future studies are warranted to determine the optimal combination ratio for this formulation. Furthermore, the action mechanisms by which KHU14 exerts its anti-inflammatory effects remain to be elucidated.

## Conclusion

The present study suggests that KHU14 exerts anti-inflammatory effects as it inhibits the production of NO and PGE_2 _in LPS/IFN-γ-stimulated peritoneal macrophages and reduces edema and the amount of infiltrated cells in animal models.

## Abbreviations

CMC: carboxymethyl cellulose; DMEM: Dulbecco's Modified Essential Medium; DMSO: dimethyl sulfoxide; DTH: delayed type hypersensitivity; IFN-γ: interferon-gamma; iNOS: inducible nitric oxide synthase; LPS: lipopolysaccharide; NO: nitric oxide; OD: optical density; PGE_2_: prostaglandine E_2_; SD: standard deviation.

## Competing interests

The authors declare that they have no competing interests.

## Authors' contributions

KSK and HIR conducted the experiments and data analysis. EKP conducted the *in vitro *experiments. KJ and HJJ prepared herbal extracts. JHK and HY assisted the *in vivo *experiments. CKH, YBC and CJR were responsible for the data analysis. HIY helped draft the manuscript. KSK and MCY prepared the manuscript. All authors read and approved the final manuscript.
